# How do cells sense oxygen?

**DOI:** 10.1007/s00424-024-03000-4

**Published:** 2024-08-05

**Authors:** Joachim Fandrey

**Affiliations:** Institut für Physiologie, Universität Duisburg-Essen, Hufelandstrasse 55 D, Essen, 45147 Germany

Oxygen — fundamental for our cells, our tissues, our organs, and the whole organism — needs to be sufficiently supplied. To that aim, cells and tissues need to sense a lack of oxygen, hypoxia, to adequately adapt to this life-threatening condition. Simply the question of how a physical condition, namely the partial pressure of oxygen (pO_2_) in cells or tissue can be translated into a biological response, has fueled scientific research for many decades. Next to fundamental work on how hypoxia stimulates respiration [10], it was the seminal finding that hypoxia in the tissue induced the production of red blood cells to compensate for hypoxia by increased oxygen capacity, and thus, transport of oxygen to the tissue. Stimulated by work from many scientists, as reviewed by Jelkmann [8], genetically modified mouse models were applied to unravel the mystery of hypoxia-induced synthesis of erythropoietin, the hormone that controls red blood cell production. Subsequent work enabled the discovery of the transcription factor hypoxia-inducible factor (HIF) controlling erythropoietin production [12]. Unravelling the way back how this protein HIF is induced by hypoxia led to the discovery of how cells sense oxygen availability through enzymes, HIF hydroxylases of which the activity is controlled by oxygen [6] [7]. These fundamental findings by Gregg L. Semenza, Sir Peter J. Ratcliffe, and William G. Kaelin Jr. were awarded with the Nobel Prize 2019 in physiology or medicine [5].

From early on in understanding the function of HIF hydroxylases, small molecule inhibitors of these enzymes were recognized. Initially used as hypoxia mimetics in experimental settings drug companies quickly recognized HIF hydroxylases as a potential pharmacological target. Ultimately, the development of small, orally available inhibitors of HIF hydroxylases led to the approval for clinical use in anemias that were caused by deficiency of erythropoietin [5]. In parallel, however, more and more cellular functions were discovered that depend on proper oxygenation and the activity of the HIF system.

This special issue of Pflugers Archive assembles a series of articles that address the fields besides classical HIF-dependent activation of target genes such as erythropoietin. Both, review articles and original contributions may shed light on potential future fields for application of prolyl hydroxylase inhibitors or areas where undesired side effects may accompany therapy with prolyl hydroxylase inhibitors.

Articles provide overviews on effects by selective deletion of one of the three hydroxylases in control of HIF [9]. While inhibitors, so far, lack absolute specificity genetic models may either provide the basis for the development of inhibitors with higher selectivity or a better understanding of tissue-specific oxygen sensing (Fig. 1). In addition, more than the three well-described HIF hydroxylases which belong to a large family of 2-oxoglutarate-dependent oxygenases are found in the tissue. Effects on metabolism by additional prolyl hydroxylases link oxygen homeostasis to metabolism [1].

**Fig. 1:**
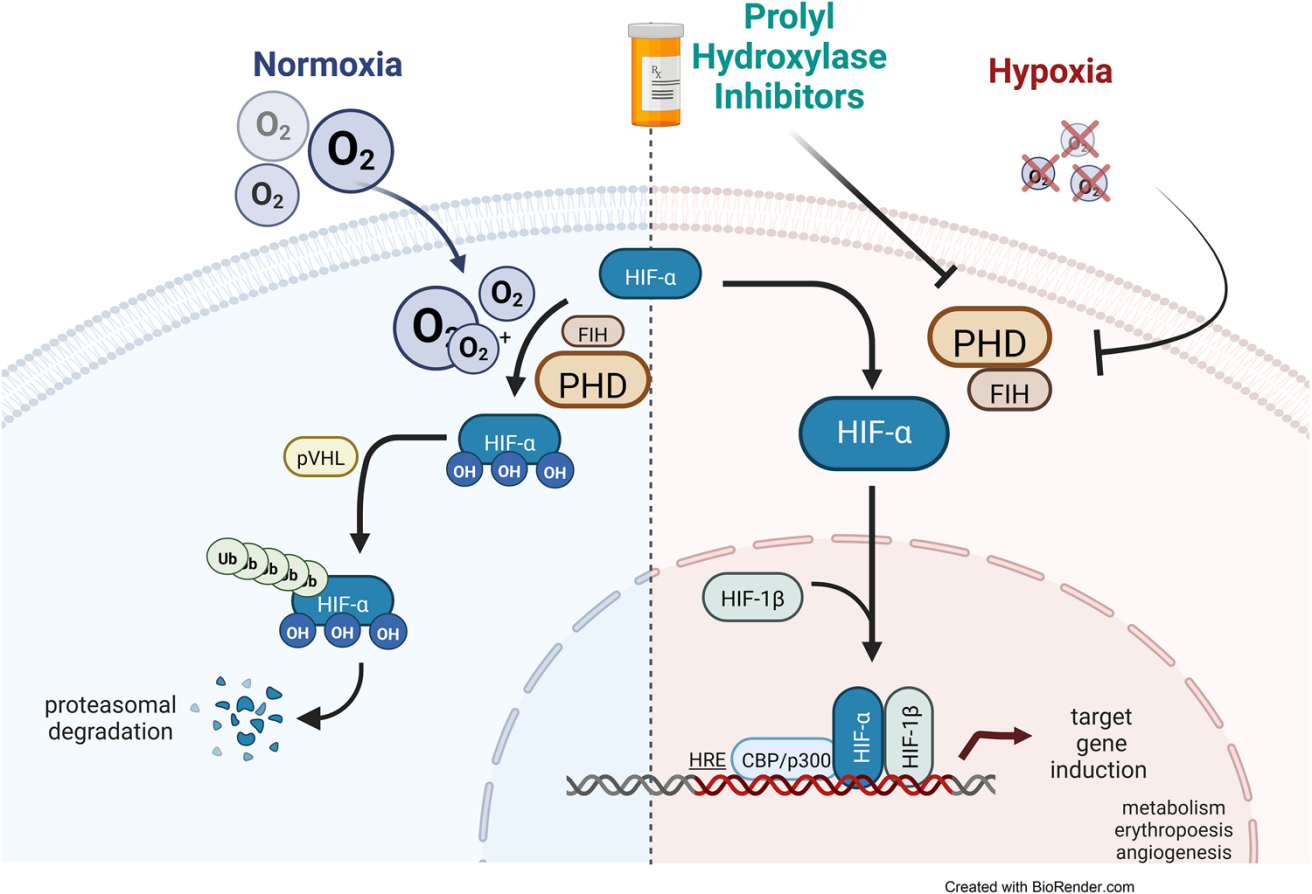
The figure was drawn by Dr. Tina Schöneberger and modified by Dr. Joachim Fandrey; created with BioRender.com

Likewise, tight control of glycolysis under hypoxia by HIF has been recognized from the very beginning of HIF research. However, while most initial work understood a role of HIF as a transcription factor in inducing glycolytic enzymes to overcome ATP shortage under hypoxia by increased glycolysis recent work from this series, here, indicates non-canonical effects of HIF in adapting glycolysis without the induction of gene expression [4]. This may also be highly relevant for situations of hypoxia in inflammation, called inflammatory hypoxia, and the interaction between the host and invading microbes.

Beyond doubt, immune cells are exposed to different areas of oxygenation, when invading into inflamed tissue [11]. Nevertheless, cells have to survive and function properly to overcome the immunological challenge. Systemic hypoxia in humans may affect the cellular HIF-dependent response of immune cells. This will be important to understand the efficiency of innate and adapted immunity under low oxygen.

Other hormones than erythropoietin depend on oxygen in their synthesis and action. Steroidogenesis has long been suspected to be influenced by hypoxia but the involvement of the HIF pathway appears to be much more complex than the initially identified control of erythropoietin expression [3]. Likewise, intercellular communication both, for cells as well as for cells with their environment, requires trafficking molecules. As complex as these trafficking mechanisms are they are often controlled by hormones and mediators, but modulated by oxygen availability. These influences may be inhibitory or stimulatory, depending on the cellular context [13].

Finally, the controversial issue of reactive oxygen species (ROS) in the process of oxygen sensing is addressed [2]. The increased production of ROS under hypoxic conditions is unanimously recognized. Effects on cellular oxygen sensing have likewise been described. In the complex situation of a pathophysiological lack of oxygen with fluctuating oxygen tensions causing hypoxia and reoxygenation, it becomes difficult to fully recapitulate this situation in an experimental model. Thus, aspects of this controversial discussion to what extent ROS are involved in oxygen sensing will be ultimately included in this special issue.

## Data Availability

No datasets were generated or analysed during the current study.
